# Dot1 promotes H2B ubiquitination by a methyltransferase-independent mechanism

**DOI:** 10.1093/nar/gky801

**Published:** 2018-09-08

**Authors:** Tibor van Welsem, Tessy Korthout, Reggy Ekkebus, Dominique Morais, Thom M Molenaar, Kirsten van Harten, Deepani W Poramba-Liyanage, Su Ming Sun, Tineke L Lenstra, Rohith Srivas, Trey Ideker, Frank C P Holstege, Haico van Attikum, Farid El Oualid, Huib Ovaa, Iris J E Stulemeijer, Hanneke Vlaming, Fred van Leeuwen

**Affiliations:** 1 Division of Gene Regulation, Netherlands Cancer Institute, 1066 CX Amsterdam, The Netherlands; 2 Division of Cell Biology, Netherlands Cancer Institute, 1066 CX Amsterdam, The Netherlands; 3 Department of Human Genetics, Leiden University Medical Center, 2333 ZC Leiden, The Netherlands; 4 Molecular Cancer Research, University Medical Center Utrecht, 3584 CG Utrecht, The Netherlands; 5 Department of Medicine, University of California, San Diego, La Jolla, CA 92037, USA; 6 UbiQ Bio B.V., 1098 XH Amsterdam, The Netherlands

## Abstract

The histone methyltransferase Dot1 is conserved from yeast to human and methylates lysine 79 of histone H3 (H3K79) on the core of the nucleosome. H3K79 methylation by Dot1 affects gene expression and the response to DNA damage, and is enhanced by monoubiquitination of the C-terminus of histone H2B (H2Bub1). To gain more insight into the functions of Dot1, we generated genetic interaction maps of increased-dosage alleles of *DOT1*. We identified a functional relationship between increased Dot1 dosage and loss of the DUB module of the SAGA co-activator complex, which deubiquitinates H2Bub1 and thereby negatively regulates H3K79 methylation. Increased Dot1 dosage was found to promote H2Bub1 in a dose-dependent manner and this was exacerbated by the loss of SAGA-DUB activity, which also caused a negative genetic interaction. The stimulatory effect on H2B ubiquitination was mediated by the N-terminus of Dot1, independent of methyltransferase activity. Our findings show that Dot1 and H2Bub1 are subject to bi-directional crosstalk and that Dot1 possesses chromatin regulatory functions that are independent of its methyltransferase activity.

## INTRODUCTION

During gene transcription, several histone modifications are deposited along transcribed regions. These post-translational modifications influence subsequent genome transactions such as transcription elongation, co-transcriptional RNA modification and processing, and the response to DNA damage and DNA repair (1–5). The introduction of co-transcriptional modifications can be mediated by interactions of the responsible modifying enzymes with RNA polymerase or with transcription elongation factors such as DRB Sensitivity Inducing Factor (DSIF) or the Paf1 complex (Paf1C; polymerase II associated factor) (6). From budding yeast to mammals, the Paf1C complex mediates the recruitment of enzymes responsible for monoubiquitination of the C-terminus of histone H2B (H2Bub1) (6–9). This bulky modification has been proposed to change the stability of nucleosomes as well as influence the structure of chromatin (10,11). H2Bub1 is a covalent modification but highly dynamic. The attachment of ub1 by the ubiquitin ligase Bre1, in conjunction with Lge1 and the ubiquitin-conjugating enzyme Rad6 in yeast, is counteracted by deubiquitinating enzymes (DUBs) Ubp8 and Ubp10, which act at distinct locations in the genome (12,13). Ubp8 is part of the deubiquitination (DUB) module of the Spt-Ada-Gcn5 acetyltransferase (SAGA) co-activator complex, which acts at promoter regions (14). Ubp10 is recruited by Sir4 to domains of silent chromatin, where it helps in maintaining a repressive chromatin state, but it also acts at euchromatic genes (12,15–17). In addition to having direct effects on chromatin structure and function, H2Bub1 is an important node for signaling to other histone modifications (11,18,19). In particular, in yeast, H2Bub1 promotes methylation of H3K4 by the Set1/COMPASS complex, methylation of H3K36 by Set2 and methylation of H3K79 by Dot1 (e.g. see (20)), and similar effects have been observed in metazoans (11,21,22).

Dot1 and methylation of H3K79 are involved in gene expression and silencing, the DNA damage response and DNA repair, and checkpoint activation in meiosis (8,23). In addition, misregulation of DOT1L in mouse and human can lead to the development of cancer (24–26). Current evidence from studies in yeast and with DOT1L *in vitro* suggest that H2Bub1 promotes the activity of Dot1 not by increased recruitment, but instead by physically corralling the enzyme into a productive binding orientation, promoting all methylation steps from unmethylated H3K79 to H3K79me1, -me2 and -me3 (19,27). As a consequence, co-transcriptional H2Bub1 deposition leads to a high methylation state (H3K79me3) in transcribed regions, whereas in intergenic regions or silent chromatin, where H2Bub1 is low, only lower methylation states (H3K79me1 and -me2) are deposited (20,28,29). Recently, several additional mechanisms of regulation of Dot1 have been described, some of which also impinge on the H2Bub-H3K79me crosstalk (20).

In addition to H3K79 methylation, Dot1 has recently been shown to possess histone chaperone activity, independent of its histone methyltransferase activity (29). However, how Dot1 and H3K79 methylation influence the structure and function of chromatin at a molecular level is still poorly understood. In order to obtain more insight into the functions of Dot1, we performed genetic interaction screens using gain-of-function alleles of *DOT1*. This approach uncovered that increased Dot1 dosage leads to increased H2Bub1 levels, and that this effect is independent of H3K79 methylation. Our findings support a revised model of crosstalk between H2Bub1 and Dot1: H2Bub1 promotes H3K79me synthesis, while the Dot1 N-terminal domain increases H2Bub1 levels, providing a potential positive feedback mechanism.

## MATERIALS AND METHODS

### Yeast strains, plasmids and growth conditions

Strains and plasmids used for the indicated figures are described in Supplementary Tables S1 and S2, respectively. Media were described previously (30). Strain NKI2378 was derived from Y7092 by first replacing the *DOT1* coding sequence by a *URA3* cassette from pR306 (31), then replacing the *URA3* cassette by the *DOT1-G401R* coding sequence derived from pFvL053 (32) and finally integrating the *TDH3* promoter amplified from pYM-N15 (33) in front of the *DOT1* gene. NKI6142 was derived from NKI6061 by replacing the *BRE1* coding sequence with the HphMX cassette from pFvL100 (34). NKI6152 was derived from NKI6061 by first replacing the *DOT1* coding sequence by a *URA3* cassette from pR306 (31), then replacing the *URA3* cassette by the *DOT1-G401A* coding sequence derived from pFvL054 (32) and finally integrating the *TDH3* promoter amplified from pYM-N15 (33) in front of the *DOT1* gene. NKI6153 was derived from NKI6061 by first replacing the *DOT1* coding sequence by a *URA3* cassette from pRS306 (31), then replacing the *URA3* cassette by the Dot1Δ2-172 fragment (amplified from pFF001, a derivative of pRS315-STR1 (34)) and finally integrating the *TDH3* promoter amplified from pYM-N15 (33) in front of the *DOT1* gene. NKI8048 and NKI8049 were derived from BY4742 by first replacing the *DOT1* coding sequence by a *URA3* cassette from pRS306 (31) and then replacing the *URA3* cassette by the *DOT1-G401V* coding sequence derived from the pRS315-DOT1-G401V plasmid described previously (35). NKI8046-49 were generated by integrating the *KanMX-TDH3* and *KanMX-TEF1* promoter cassette amplified from pYM-N14 and pYM-N18, respectively (33) in front of the *DOT1* gene. NKI2509, NKI2544, NKI2512, NKI2515, NKI2518 and NKI2521 were generated by replacing the *DOT1* coding sequence with the NatMX cassette from pFvL99 (34). NKI2510, NKI2545, NKI2513, NKI2516, NKI2519, NKI2522, NKI2528 and NKI2566 were generated by integrating the *NatNT2-TDH3* promoter cassette amplified from pYM-N15 (33) in front of the *DOT1* gene. pFvL006 was derived from pFvL018 to generate pTCG-DOT1-254-582. pFvL019 was derived from pFvL018 by deletion of a 1.3 kb NruI–BamHI fragment to generate pTCG-DOT1-1-172. Strains NKI3027 and NKI3028 were derived from UCC7315 by replacing plasmid pCS1 with pRG422 and pRG423, respectively (36). Strains NKI2563 and NKI2564 were derived from UCC6288 by replacing plasmid pCS1 with pRG422 and pRG423, respectively (36). Strain UCC6288 was derived from UCC7315 by replacing the *UBP8* coding sequence by a *KanMX* cassette from pRS400 (31). Strain NKI2527 was derived from Y7092 by replacing the *PAF1* coding sequence by a *KanMX* cassette from pRS400 (31). NKI4748 was isolated from the Epi-Decoder library described elsewhere (37).

### Genetic interaction analysis

High-throughput genetic interactions were determined based on epistatic miniarray profiling (E-MAP) (38). Double mutants were constructed using the RoToR from Singer Instruments (Watchet, UK) and the synthetic genetic array (SGA) technology (20,35,39). Static growth scores were computed as previously described in (40). The library of ∼1400 deletion- and decreased abundance by mRNA perturbation (DAmP)-mutants has been described previously (40).

### Transcriptome analysis

Messenger RNA expression profiles of wild-type (WT) (BY4742), *dot1Δ* (NKI3002), Dot1-OE (NKI8046 and NKI8047) and Dot1-OE-G401V (NKI8048 and NKI8049, overexpressing catalytically inactive Dot1 in the absence of endogenous WT DOT1) were generated as part of a large and uniform collection of deletion and perturbation mutants (41,42). Expression profiling and data analysis were performed as described previously (41,42).

### Protein detection by immunoblot and antibodies

For immunoblotting, strains were grown to mid-log phase (OD660 0.6–0.9). Samples of 2 × 10^8^ cells were harvested and washed with Tris-EDTA (TE; 10 mM Tris pH 8, 1 mM ethylenediaminetetraacetic acid (EDTA)) containing 0.2 mM phenylmethane sulfonyl fluoride (PMSF). Cell pellets were stored at −80°C until further processing, but at least 30 min. Whole-cell extracts were prepared in SUMEB (1% sodium dodecyl sulfate (SDS); 8 M urea; 10 mM 3-(N-morpholino)propanesulfonic acid, pH 6.8; 10 mM EDTA; 0.01% bromophenol blue) containing protease inhibitors (1 mM PMSF, 1 mM dithiothreitol, 5 mM benzamidine, 1 μg/ml pepstatin, 1 μg/ml leupeptin) by bead beating. The resulting lysate was incubated for 10 min at 65°C and subsequently clarified by centrifuging 5 min at 21 × g. Before immunoblotting, 4–10 μl of lysate (∼2 × 10^6^ cells) was separated on a polyacrylamide gel (16% for histone H3 and H2B, 10% for Pgk1, Dot1, FLAG and TAP). Separated proteins were transferred to a 0.45-μm nitrocellulose membrane for 1 (H3 and H2B) or 2 h (Pgk1, Dot1, FLAG, TAP) at 1 A. Membranes were blocked with phosphate-buffered saline (PBS) containing 2% or 5% Nutrilon (Nutricia) for 1 h, and first antibody incubations (dilutions see below) were performed overnight at 4°C in 4 ml Tris-buffered saline containing 0.05% Tween-20 (TBST) with 2% Nutrilon. After washing three times in TBST, secondary antibody incubation was performed in TBST with 2% Nutrilon and LI-COR Odyssey IRDye 800CW antibody at 1:10 000 for 45 min at room temperature in the dark followed by 10 min washes twice in TBST and once in PBS. Membranes were scanned using an LI-COR Odyssey IR Imager (Biosciences) and analyzed using Image Studio 2.0 (LI-COR). For density scans, the signal was the sum of the individual pixel intensity values for a shape minus the product of the median intensity values of the pixels in the background (with a border width top/bottom of 3) and the total number of pixels enclosed by the shape (Area): Signal = Sum − (Background × Area). Primary antibodies and their dilutions used in this study are Pgk1 (459250, Invitrogen, RRID:AB_221541; 1:4000), Histone H2B (39238, Active Motif, RRID:AB_2631110; 1:2000), Flag (M2 F3165, Sigma, RRID:AB_259529; 1:4000), histone H3 (RRID:AB_2631108 (43); 1:2000), TAP (CAB1001, ThermoFisher Scientific, RRID:AB_10709700; 1:1000), Dot1-C (RRID:AB_2631109 (36); 1:2000), yDot1 (#144682, this manuscript, RRID:AB_2737408; 1:1000), yH2BK123ub1 (this manuscript, 152107, Ximbio, RRID:AB_2737407; 1:5000 (10 mg/ml)), H3K79me1 (RRID:AB_2631105 (36); 1:1000), H3K79me2 (04–835, Millipore, RRID:AB_1587126; 1:2000) and H3K79me3 (RRID:AB_2631107 (36); 1:1000). Secondary antibodies used are IRDye 800CW goat anti-Mouse igg (0.5 mg) 926-32210 Li-COR (RRID:AB_621842) and IRDye 800CW goat anti-Rabbit igg (0.5 mg) 926-32211 Li-COR (RRID:AB_621843).

### Chromatin immunoprecipitation (ChIP)

For chromatin immunoprecipitation (ChIP), cells were grown to mid-log phase in Yeast Extract Peptone Dextrose media (YEPD). Samples of 1−3 × 10^9^ cells were taken, fixed for 10 min in 1% formaldehyde, and washed with cold TBS. Pellets were stored in 2-ml screw-cap tubes at −80°C until further processing. Cells in 2 ml screw-cap tubes were disrupted in 400 μl of breaking buffer (100 mM Tris, pH 7.9; 20% glycerol; protease inhibitor cocktail EDTA-free; Roche) with 400 μl of zirconia/silica beads with a Bead beater (Biospec Products) for 2 × 2 min in a cold aluminum rack at 4°C. Lysis was at least 70%, as determined by microscopy. Lysates were diluted with 1 ml of FA buffer (50 mM 4-(2-hydroxyethyl)-1-piperazine-ethanesulfonic acid–KOH (HEPES–KOH), pH 7.5; 140 mM NaCl), 1 mM EDTA; 1% Triton X-100; 0.1% Na-deoxycholate; protease inhibitor cocktail EDTA-free. The mixture was centrifuged for 1 min at 21 × g at 4°C, and the pellet was washed once more with FA buffer. The pellet was resuspended in 450 μl of FA, divided over two 1.5 ml Bioruptor Microtubes with Caps (Diagenode #C30010016) and sonicated for 6−7 min in a Bioruptor Pico (Diagenode) with 30 s on–off cycles on high power. Lysates were cleared by centrifugation for 5 min at 4°C at 21 000 × g. Supernatant containing chromatin was transferred to a 1.5-ml tube and 1 ml of FA was added to samples. The chromatin solution was centrifuged for 15 min at 21 000 × g at 4°C; the supernatant was transferred to a new 1.5-ml tube and stored at −20°C. Magnetic Dynabeads coupled with Protein G (Life Technologies) were incubated in PBS containing 5 mg/ml bovine serum albumin with antibody for at least 4 h at 4°C. The following antibodies were used for ChIP: Histone H2B (39238, Active Motif, RRID:AB_2631110), yH2BK123ub (this manuscript). Subsequently, 400 μl of soluble chromatin was added to 40 μl prepared Dynabeads and incubated rotating overnight at 4°C and 1 ml of FA buffer was added and samples were incubated rotating for 5 min at room temperature. The beads were washed twice with each of the buffers FA, FA-HS (50 mM HEPES–KOH, pH 7.5; 500 mM NaCl; 1 mM EDTA; 1% Triton X-100; 0.1% Na-deoxycholate) and RIPA (10 mM Tris, pH 8; 250 mM LiCl; 0.5% NP-40; 0.5% Na-deoxycholate; 1 mM EDTA). Finally, the beads were washed once with TE (10 mM Tris, pH 8; 1 mM EDTA). Then 100 μl of elution buffer (50 mM Tris, pH 8; 10 mM EDTA; 1% SDS) was added to the samples and incubated for 10 min at 65°C. Subsequently, the samples were centrifuged 1 min at 21 × g and 80 μl of supernatant was collected. Then, 70 μl of TE was added to samples and crosslinks were reversed in 0.625 mg/ml ProtK and 3 μg/ml RNaseA incubated for 1 h at 50°C and subsequently overnight at 65°C. For input samples, 40 μl of chromatin solution was combined with 60 μl elution buffer and 70 μl of TE and treated in the same manner as ChIP samples to reverse crosslinks. DNA was purified by using the QIAquick PCR Purification Kit (Qiagen). Alternatively, for some qPCR samples, DNA was extracted by using Chelex-100 resin (Bio-Rad) (44,45).

### Quantitative PCR

All qPCR analyses were performed considering the general MIQE guidelines (46) and using the specific conditions described below and the primers described in Supplementary Table S3. Quantitative real time-PCR (qPCR) was performed with SensiFAST Sybr No-Rox Mix 2x (Bioline) according to the manufacturer’s manual. IP and input samples were diluted 100 times before analyzing by qPCR on a LightCycler 480 II (Roche). Cycling parameters were as follows: 1 cycle pre-incubation: 2 min 50°C, 10 min 95°C; 50 cycles amplification: 15 s 95°C (4.4°C/s), 60 s 60°C (2.2°C/s) acquisition mode: single; 1 cycle melting curve: 15 s 95°C (4.4°C/s), 60 s 60°C (2.2°C/s) 95°C (0.11°C/s) acquisition mode: continuous.

### ChIP-sequencing and data analysis

Samples were pooled equimolarly and subjected to sequencing on an Illumina HiSeq2000 machine in a single-read 65 bp run. Reads were mapped to the *Saccharomyces cerevisiae* reference genome R64-2-1 with BWA version 0.6.1 and filtered for mapping quality below 37 (47). Each read was extended to 150 bp. Each sample was normalized for the sequencing depth by converting to Reads per Genomic Content (RPGC) with DeepTools (48). This was done by first calculating the sequencing depth: (total number of mapped reads * fragment length)/effective genome size (12.1 × 106 bp). Then, the coverage was multiplied by the scaling factor: 1/sequencing depth to get RPGC. Data from the biological duplicates were found to be similar and the datasets were merged for further analyses. Metagene plots and heatmaps were generated with custom scripts in R/Bioconductor (49). Reads were either aligned in a window of −500 to 1 kb around the TSS or +/− 2 kb from the center of each gene. Genes that contain a coverage of 0 or an average coverage in the first 500 bp below 0.5 were filtered out (5006 out of 5134 genes remained). Genes were obtained from yeastgenome.org, and transcription data were obtained from McKnight *et al.* (2015) (50). The small-scale Epi-ID experiment was performed as described in (20), using the same ChIP sample as described in that paper.

## RESULTS

### Genetic interactions between increased dosage of Dot1 and loss of SAGA-DUB function

**Figure 1.**
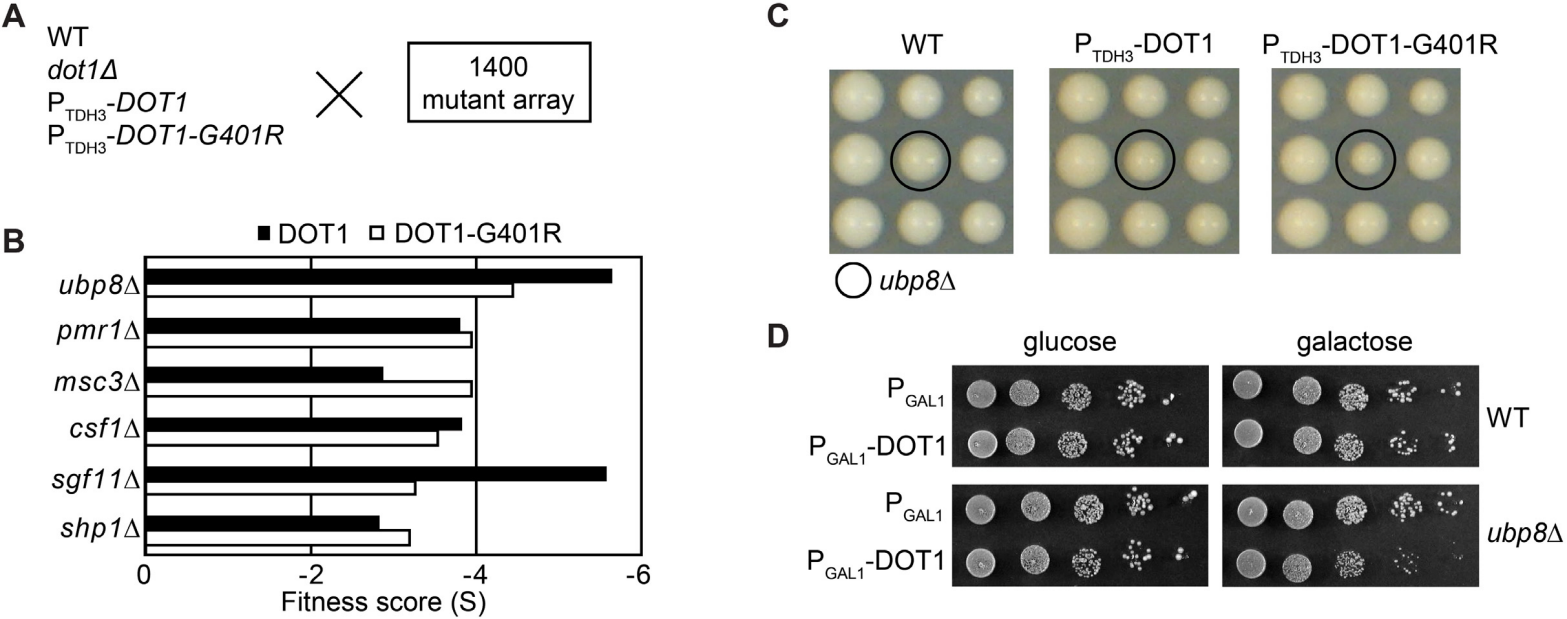
Genetic interactions between Dot1 overexpression and loss of SAGA-DUB suggest a common function of Dot1 and Ubp8. (**A**) Strains expressing endogenous Dot1 (WT), no Dot1 (*dot1Δ*), high levels of Dot1 (*P_TDH3_-DOT1*) or high levels of catalytically inactive Dot1 (*P_TDH3_-DOT1-G401R*) were crossed to an array of ∼1400 mutant strains and examined for fitness. (**B**) Fitness score (*S*) of mutants most affected by overexpression of Dot1 where *S* > 0 represents better growth than expected and *S* < 0 worse growth than expected. (**C**) Images of plates as an example of the colony fitness defect of the *ubp8Δ* strain. (**D**) Validation of the negative genetic interactions between *ubp8Δ* and overexpression of Dot1 from a galactose-inducible *GAL1* promoter on a multicopy (2 μ) plasmid (+) or using an empty vector control (-).

Systematic screens for genetic interactions have been used to uncover gene functions and functional relationships. To identify unknown functions of Dot1, we determined the genetic interaction profiles of a set of *DOT1* alleles in *S. cerevisiae* using SGA analysis of a library containing ∼1400 knockouts and decreased abundance by mRNA perturbation (DAmP) mutants, representing a range of biological processes with a bias toward nuclear functions (40). Since gene deletions and increased gene dosage alleles often uncover different biological functions (51), we analyzed a *dot1Δ* strain (lacking all H3K79 methylation), Dot1 overexpression under control of the strong *TDH3*/GADPH promoter (Dot1-OE; showing nearly exclusively H3K79me3 at virtually all H3 molecules) and a catalytically inactive Dot1-G401R mutant overexpressed by the *TDH3* promoter in the absence of endogenous WT Dot1 protein (Dot1-G401R-OE). The *DOT1* alleles by themselves had no effect on cell growth and mating efficiency, and showed minimal effects on the cellular transcriptome by expression profiling (Supplementary Table S4). For the SGA analysis, we crossed the *DOT1* alleles into the mutant library and determined the genetic interaction maps of the *DOT1* alleles using a series of previously used query genes (40). This resulted in interaction scores (*S*) where a positive score (*S* > 0) represents better growth than the expected double mutant fitness and a negative score (*S* < 0) worse than expected (Figure 1A).

Deletion of *DOT1* showed very few genetic interactions, as we observed previously (35). Overexpression of Dot1 resulted in a different interaction profile. Among the strongest negative interactions of increased Dot1 dosage (S<3) were deletion of *UBP8* and *SGF11*, encoding two of the three subunits of the deubiquitination module (DUB) of the SAGA co-activator complex present in the library used (Supplementary Table S5; Figure 1B and C). These interactions were found in untreated cells as well as in cells exposed to DNA damaging stress (Supplementary Table S5). Non-DUB SAGA subunits did not show negative interactions (see Supplementary Table S5), indicating that the phenotype was not related to the general integrity of the SAGA complex but specific to the DUB module.

Therefore, we next focused on the cause of the genetic relationship between SAGA-DUB and increased Dot1 expression. We previously showed that some genetic interactions in SGA analysis can be caused by synthetic loss of silencing and thereby loss of mating-type maintenance (35). For example, the genetic interaction between *dot1Δ* and *sir1Δ* in SGA screens is caused by loss of mating-type maintenance and therefore specific for the SGA conditions; synthetic sickness is not observed in standard growth conditions (35). Synthetic loss of mating type in *dot1Δsir1Δ* strains was confirmed in the screen described here and was also observed when *sir1Δ* was combined with Dot1-OE or Dot1-G401R-OE (Supplementary Table S5 and Supplementary Figure S1A). We note that deletion of *DOT1* or overexpression of WT or inactive Dot1 by itself did not lead to mating defects based on transcriptome analysis (Supplementary Table S4) and based on the fact that all the query strains efficiently mated to the mutant collection. In contrast to genetic interactions caused by synthetic mating defects, the genetic interaction between Dot1-OE and SAGA-DUB inactivation could be validated under non-SGA conditions and using conditional expression constructs: overexpression of Dot1 from an inducible *GAL1* promoter on a high-copy plasmid resulted in reduced colony growth in a *ubp8Δ* background when compared to an empty plasmid, whereas no effect on growth was observed in WT strains (Figure 1D). Therefore, the genetic interaction observed in the SGA screen was not caused by mating-type defects and occurred under two independent conditions of Dot1 overexpression.

### The Dot1–SAGA-DUB genetic interaction is independent of H3K79 methylation

A negative genetic interaction can be indicative of two genes affecting the same process by independent pathways or mechanisms. Following this logic, we first asked whether the synthetic sickness could be explained by extremely high H3K79 methylation levels. The Dot1-OE strain already has very high levels of H3K79me3, leaving only a small amount of histone H3 with lower methylation states (34,36,52,53). The absence of Ubp8, leading to more H2Bub1, could lead to further loss of the lower methylation states (20). To test this idea, we examined the genetic interactions of the P_TDH3_-DOT1-G401R allele, which leads to overexpression of a Dot1 protein that cannot bind its co-factor SAM and can therefore no longer methylate H3K79 (34,36,52,53). Importantly, in this Dot1-G401R-OE strain, no WT copy of *DOT1* was present. The genetic interaction profiles of overexpressed WT and Dot1-G401R were very similar and both showed negative interactions with *ubp8Δ* and *sgf11Δ* (Supplementary Table S5; Figure 1B and C). Therefore, the synthetic sickness was not caused by extremely high H3K79me levels. In agreement with this, extremely high H3K79me2/H3K79me3 levels induced by ectopic expression of *Trypanosoma brucei* DOT1A or DOT1B proteins in yeast did not have any obvious effects on growth rate either (52). Furthermore, unlike overexpressed WT Dot1, overexpressed catalytically dead (G401R) or compromised (G401A) Dot1 did not disrupt silencing of telomeric reporter genes (Supplementary Figure S1B and C), confirming that the synthetic sickness did not involve loss of silencing.

### Dot1 promotes H2B ubiquitination

**Figure 2.**
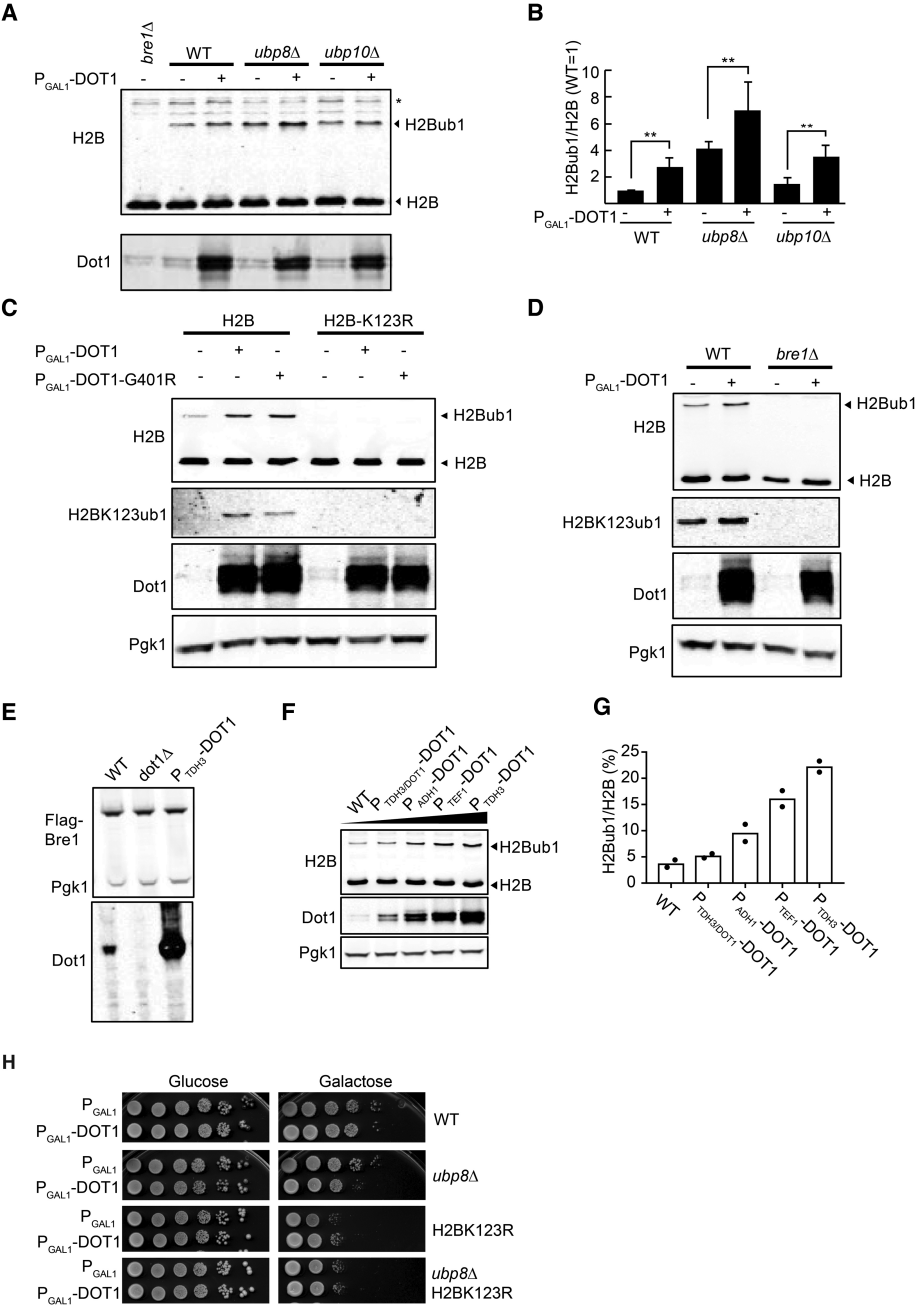
Overexpression of Dot1 promotes H2BK123ub1 independent of deubiquitinases Ubp8 and Ubp10. (**A**) Immunoblot analysis showing monoubiquitination of H2B in WT, *ubp8Δ* and *ubp10Δ* strains with or without overexpression of Dot1 using an inducible *GAL1* promoter on a multicopy (2 μ) plasmid. H2Bub1 can be detected by the slower migrating band using H2B antibodies. The asterisk indicates a non-specific band, shown as a loading control. The Dot1 antibody was used to show the Dot1 overexpression. (**B**) Quantification of the immunoblot shown in (A) (H2Bub1/H2B relative to WT) and biological replicates thereof (*N* = 3 +/- SD). Statistical significance as determined by an unpaired *t*-test is indicated by the asterisks (*P < 0.1, **P < 0.05). (**C**) Immunoblot analysis of strains harboring either WT H2B or H2B-K123R, with either overexpressed Dot1 or Dot1-G401R using an inducible *GAL1* promoter on a multicopy (2 μ) plasmid. A site-specific antibody demonstrates that the site of ubiquitination is H2B-K123. Pgk1 was used as a loading control. (**D**) Immunoblot analysis of WT and *bre1Δ* cells with or without overexpression of Dot1 using an inducible *GAL1* promoter on a multicopy (2 μ) plasmid. (**E**) Dot1 overexpression does not affect the expression level of Bre1, the main factor responsible for H2BK123ub1. An N-terminal FLAG tag was used to preserve the E3 activity. (**F**) Immunoblot analysis of strains expressing increasing amounts of Dot1 shows that Dot1 promotes H2BK123ub1 in a dose-dependent manner. (**G**) Quantification of the immunoblot shown in (F) (mean and individual data points of two biological replicates). (**H**) Suppression of synthetic sickness of Dot1-OE and *ubp8Δ* by an H2BK123R mutation. Dot1 was overexpressed in the strains indicated from a galactose-inducible *GAL1* promoter on a multicopy (2 μ) plasmid (P_GAL1_-DOT1) and an empty vector was used as a negative control (P_GAL1_). Strains were spotted in a 10-fold dilution series and were pre-grown for 24 h in the carbon source indicated.

Having excluded H3K79 hypermethylation as the cause of the synthetic sickness, we next investigated the possibility that the shared process that SAGA-DUB and overexpressed Dot1 regulate is ubiquitination of histone H2B. On immunoblots, monoubiquitination of H2B results in a slower migrating band that can be readily detected using H2B antibodies. Indeed, immunoblot analysis of histone H2B showed that increased Dot1 expression led to higher levels of H2Bub1 (Figure 2A and B). This was not caused by higher H3K79 methylation levels because overexpression of Dot1-G401R also led to more H2Bub1 (Figure 2C and Supplementary Figure S2A) and expression of TbDOT1A or TbDOT1B had no effect (Supplementary Figure S2A). Importantly, overexpression of Dot1 in a *ubp8Δ* strain further elevated the already high levels of H2Bub1 in this background (Figure 2A). Even though the combined effect of Dot1-OE and *ubp8Δ* was additive rather than synergistic, these findings support the idea that the synthetic sickness of overexpressed Dot1 and *ubp8Δ* was caused by a very high level of H2Bub1. It also shows that the mechanism through which Dot1 promotes H2Bub1 does not involve inhibition of Ubp8. To obtain more support for the idea that Ubp8 and Dot1 affect the same process, we investigated whether Dot1 and Ubp8 regulate ubiquitination on the same site of H2B, i.e. H2BK123ub1. Two lines of evidence suggested that Dot1 indeed promotes specific ubiquitination of H2BK123. First, Dot1-dosage dependent H2B monoubiquitination was abrogated by an H2BK123R mutant that cannot be ubiquitinated on H2BK123 (Figure 2C). Second, the effect of Dot1 overexpression on H2Bub1 required the H2BK123-ubiquitin ligase Bre1 (Figure 2D). However, Dot1 overexpression did not affect Bre1 protein levels (Figure 2E) and it did not affect the mRNA expression of Bre1 or other factors known to control H2BK123ub1 levels in the cell (genes marked in Supplementary Table S4). Ubp8 mRNA and protein levels were also unaltered (Supplementary Table S4 and Supplementary Figure S2B). A Dot1-OE-induced increase in H2Bub1 was still observed in a *paf1Δ* background, in which basal H2BK123ub1 levels are lower than in WT cells, suggesting that Dot1 acts downstream of Paf1C to stimulate the activity of Bre1 (Supplementary Figure S2C; ***P < 0.001).

To directly demonstrate the site of ubiquitination, we raised a site-specific monoclonal antibody against yeast H2BK123ub1 using a synthetic Ub-polypeptide antigen based on amino acids 115–130 of yeast H2B. This antibody detects H2BK123ub1 but does not react with unmodified H2B, free ubiquitin, or ubiquitin covalently attached to H2A (Supplementary Figure S2D). Immunoblot analysis of bulk histones showed that overexpression of Dot1 or Dot1-G401R led to increased H2BK123ub1 (Figure 2C). Finally, the effect of Dot1 on H2BK123ub1 was not just observed at very high levels of overexpression. Analysis of a series of *DOT1* alleles ranging from WT to very high expression showed a dose-dependent enhancement of H2BK123ub1 by Dot1 (Figure 2F and G). On the other hand, deletion of *DOT1* did not lead to lower H2BK123ub1 levels (Supplementary Figure S2E), suggesting that in WT cells, the stimulatory effect of Dot1 on H2BK123ub1 is redundant with other mechanisms. Together, these findings demonstrate that increased Dot1 dosage leads to increased H2BK123ub1 in a dose-dependent manner, suggesting that the known crosstalk from H2Bub1 to H3K79me is also subject to a reverse crosstalk from Dot1 to H2Bub1.

Finally, having established that Dot1 overexpression leads to an increased level of H2B ubiquitination and that this level is even higher in the absence of Ubp8, we tested whether this very high level of H2Bub1 is responsible for the genetic interaction of the Dot1-OE *ubp8Δ* double mutant. Indeed, introduction of an H2BK123R mutation, which eliminates all H2BK123ub1, suppressed the synthetic sickness while Dot1 protein overexpression levels in the Dot1-OE *ubp8Δ* strain were not affected by the H2BK123R mutation (Figure 2H and Supplementary Figure S2F).

### Dot1 overexpression promotes H2BK123ub1 across the genome

**Figure 3.**
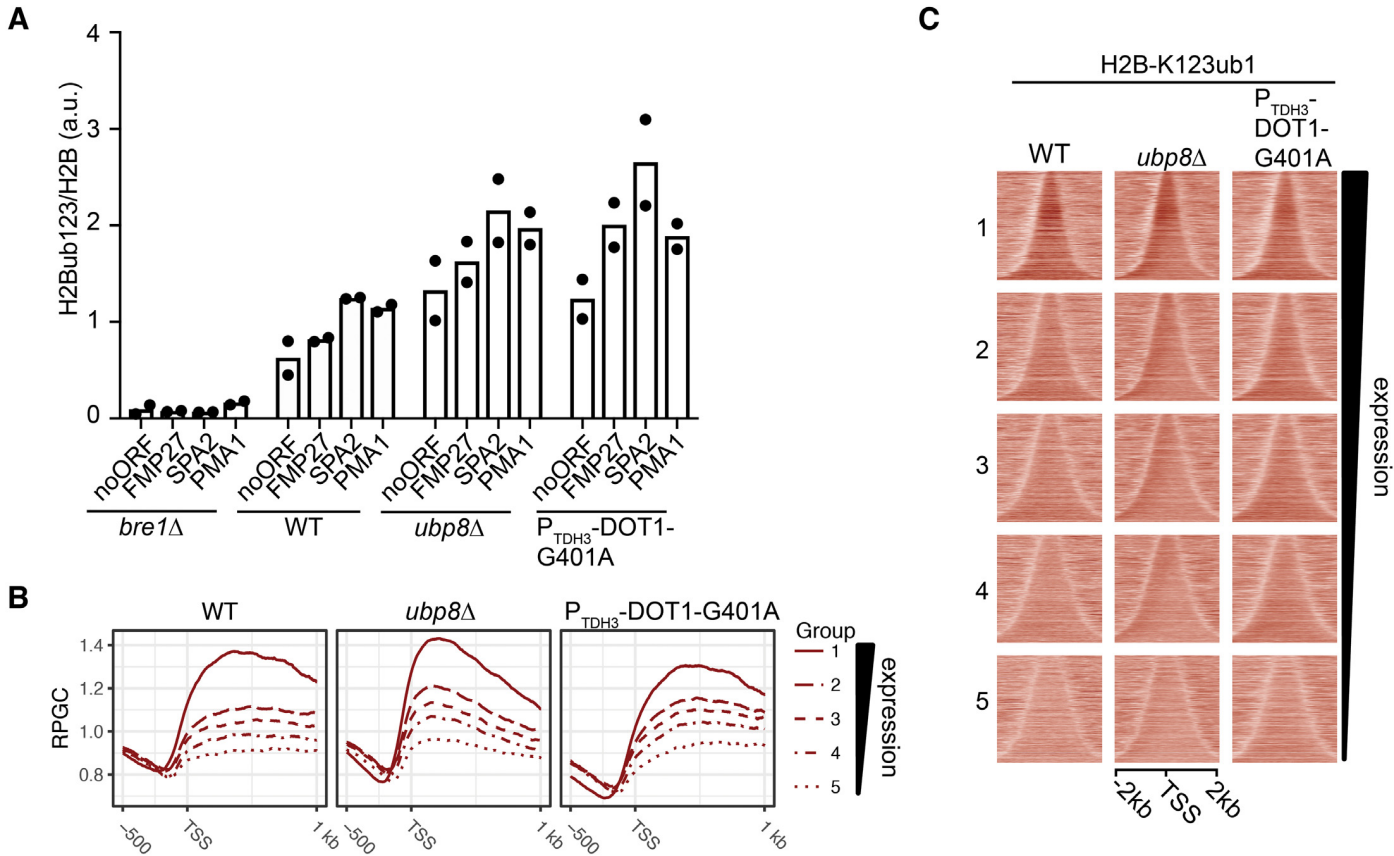
Effects of Dot1 overexpression and loss of Ubp8 on H2BK123ub1 in chromatin. (**A**) ChIP-qPCR analysis of H2BK123ub1 relative to H2B in transcribed regions in *bre1Δ*, WT, *ubp8Δ* and Dot1-G401A overexpression under the control of the strong *TDH3* promoter (mean and individual data points of two biological replicates). (**B**) Metagene plots of H2BK123ub1 ChIP-seq in WT, *ubp8Δ* and Dot1-G401A showing the average H2BK123ub1 pattern around the transcription start site. Colored lines represent five different groups based on gene expression level, from high (group 1) to low (group 5) expression. (**C**) Heatmaps of read-depth normalized H2BK123ub1 ChIP-seq counts showing the H2BK123ub1 signal in the five different gene expression groups indicated in B. Genes within each subgroup were ranked on gene length and centered on the gene midpoint.

Our results show that Dot1 can promote monoubiquitination of H2BK123 by Bre1. To obtain more insight into the role of Dot1 in promoting H2B ubiquitination, we investigated the changes in H2BK123ub1 upon Dot1 overexpression by ChIP. ChIP-qPCR with the H2BK123ub1 antibody confirmed that the H2BK123ub1 levels were increased in a *ubp8Δ* strain, and indicated that overexpression of a catalytic mutant of Dot1 (Dot1-G401A) led to an H2BK123ub1 increase in intergenic and transcribed regions as well (Figure 3A). Multiplexed ChIP, using our previously developed ChIP-Barcode-Seq Epi-ID technology (20), confirmed the increase in H2BK123ub1 at a barcoded reporter gene in a *ubp8Δ* strain as well as the strain overexpressing Dot1 (Supplementary Figure S3A).

We next performed ChIP-sequencing to analyze the pattern of H2BK123ub1 in Dot1 overexpression strains and how it compared to WT and *ubp8Δ* strains. No major changes in H2B occupancy were observed in the mutant strains (Supplementary Figure S3B). Figure 3B shows the average H2BK123ub1 pattern across genes at different expression levels. As expected, in WT cells, the average H2BK123ub1 levels correlated with gene expression and peaked across transcribed regions. In the Dot1-OE strain, the increase in H2BK123ub1 levels that can be observed on immunoblots (Figure 2) did not affect the general distribution of H2BK123ub1 (Figure 3B and C). The increase in H2BK123ub1 occurred across transcribed regions, following the pattern of endogenous H2BK123ub1 as well as that of H3K79me2/3 laid down by Dot1 (54). Inspection of different gene expression groups showed that in the Dot1-OE strain, the difference between highly (group 1) and lowly (group 5) expressed genes was reduced, indicating that lowly transcribed genes with low H2BK123ub1 in WT cells gained relatively more H2BK123ub1 than highly transcribed genes already marked with higher levels of H2BK123ub1 in WT cells. These observations together show that Dot1-OE generally enhances the WT H2BK123 ubiquitination activity, i.e. at transcribed regions where it normally engages in H3K79 methylation, and especially affects lowly transcribed genes that harbor low endogenous levels of H3BK123ub1. In contrast, the *ubp8Δ* strain showed enrichment of H2BK123ub1 at the 5′ end of genes (Figure 3B and C) consistent with previous observations (12), and did not affect lowly transcribed genes more than highly transcribed genes. The observed activity of Ubp8 at the beginning of genes is in agreement with the targeting of the SAGA-DUB complex to promoter regions (12,14). Together, the ChIP-seq results show that Dot1-OE and loss of Ubp8 stimulate H2BK123ub1 at different locations in the genome, further strengthening the idea that they stimulate H2BK123ub1 by distinct mechanisms.

### The N-terminus of Dot1 promotes ubiquitination of H2BK123

**Figure 4.**
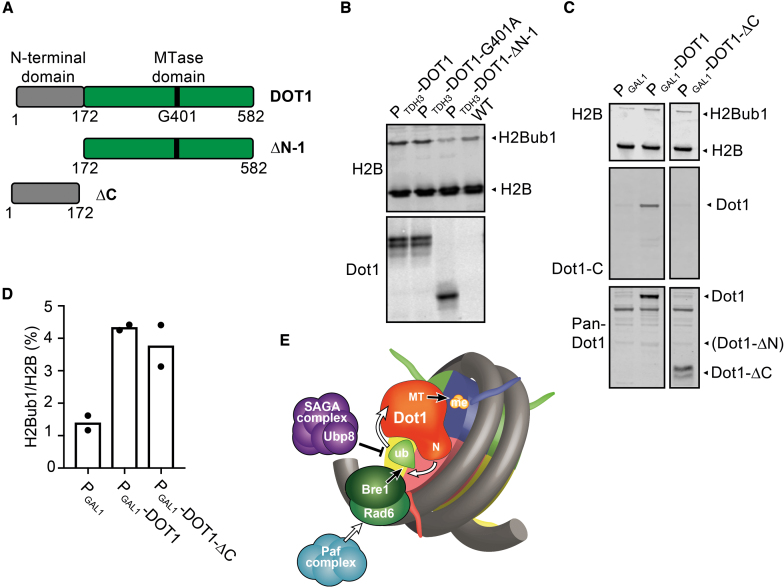
The N-terminus of Dot1 is necessary and sufficient to promote H2Bub1. (**A**) Schematic representation of the yeast Dot1 protein and different mutants used in this study. (**B**) Deletion of the Dot1 N-terminal part (DOT1-ΔN-1) abolished the effect of Dot1 on H2Bub1 without altering the expression level of constitutively overexpressed Dot1. (**C**) Inducible overexpression in galactose media of the N-terminus alone (Dot1-ΔC) was sufficient to promote H2Bub1. The lane with DOT1-ΔC-terminal originates from the same blot. The pan-Dot1 antibody was raised against full-length Dot1, but preferentially recognizes the N-terminus (Supplementary Figure S4). (**D**) Quantification of the immunoblot shown in (C) (mean and individual data points of two biological replicates). (**E**) A model for the mutual crosstalk between Dot1 and H2Bub. The catalysis of (de)modifying reactions is indicated by a black arrow or bar-headed line. Stimulation is visualized by white arrows. In short, H2BK123ub1 promotes H3K79 methylation by Dot1 and the N-terminus of Dot1 promotes ubiquitination of H2BK123. The latter stimulation is independent of deubiquitination by Upb8 and the recruitment of Bre1/Rad6 by the Paf complex, and is thus likely to directly act on ubiquitination by Bre1.

Finally, we analyzed a series of Dot1 mutants to obtain more information on the domains of Dot1 responsible for the crosstalk. Yeast Dot1 contains an N-terminal part involved in nucleosome binding and the chaperone function of Dot1, and a C-terminal part harboring the methyltransferase domain (Figure 4A) (29,55,56). Deletion of the N-terminal domain (Δ1–172) abolished the effect of Dot1 on H2BK123ub1 while the remaining C-terminal part of the protein was still expressed at high levels in this mutant (Figure 4B). Furthermore, the N-terminus alone, lacking the methyltransferase domain, could still promote H2B ubiquitination (Figure 4C and D). Therefore, the N-terminal domain of Dot1 was necessary and sufficient for the reverse crosstalk from Dot1 to H2BK123ub1.

## DISCUSSION

The H3K79 methyltransferase activity of Dot1 in yeast and other organisms is regulated by the attachment of a ubiquitin moiety to the C-terminus of H2B. Here we show that Dot1 in yeast can in turn promote ubiquitination of H2B. This suggests that there is a mutual crosstalk between Dot1 and H2Bub1 that can fine-tune the levels of H2Bub1 and H3K79me in transcribed regions where the ubiquitination machinery and Dot1 primarily act. The mutual crosstalk in principle provides a possibility for a positive feedback loop in which H2Bub1 can promote Dot1 activity and thereby further stimulates H2Bub1. However, this may not be a general scenario because current evidence suggests that H2Bub1 promotes the Dot1 catalytic activity but not the binding to nucleosomes (19,27). At sites where Dot1 is bound or recruited, however, Dot1 may promote its own activity by enhancing H2Bub1 synthesis, which will enhance subsequent H3K79 methylation events by the distributive Dot1 enzyme (36).

The higher H2Bub1 levels caused by increased Dot1 dosage provide several insights into the functions of Dot1 and H2Bub1. First, we previously showed that forced recruitment of catalytically inactive Dot1 to domains of silent chromatin in yeast leads to desilencing and subnuclear relocalization (53). The crosstalk from Dot1 to H2Bub1 that we describe here suggests that these chromatin rearrangements induced by Dot1 might be mediated by increased H2Bub1, which in turn can affect chromatin structure and downstream histone modifications. Thus, methyltransferase-independent functions of Dot1 affect biochemical features of chromatin and may also affect the functional output. Second, mutants that affect H2Bub1 levels generally have profound effects. Mutants of the SAGA-DUB complex, the Bre1/Rad6/Lge1 ubiquitination machinery or the upstream regulatory PAF1 complex, and mutants of H2BK123 all show problems with gene expression as well as various fitness defects (11,18,21,42). Dot1 dosage provides an independent means of manipulating H2Bub1 levels. It surprisingly reveals that increasing overall H2Bub1 levels by Dot1 to a similar extent as in a *ubp8Δ* strain affects the expression of only very few genes (see Supplementary Table S4) and shows no obvious fitness defects, even when combined with other mutations (Supplementary Table S5), except for synthetic silencing defects (Supplementary Table S5 and Supplementary Figure S1A). Thus, even though H2Bub1 in chromatin correlates well with transcription level, the absolute level of H2Bub1 does not seem to be a strict determinant of gene expression. Since the distribution of H2Bub1 across genes is not much altered upon Dot1 overexpression but is altered in *ubp8Δ* (Figure 4), it is possible that the relative amount of H2Bub1 across genes is a factor that influences transcriptional output.

How does Dot1 affect H2Bub1? Our results show that the N-terminus of Dot1 is necessary and sufficient to promote H2Bub1. This part of Dot1 has been shown to bind to nucleosomes *in vitro* and to support full activity of Dot1 *in vitro* and *in vivo*. Moreover, the N-terminus of Dot1 can act as a derepressor and desilencer when targeted to yeast heterochromatin (53) and was recently shown to be required for a methyltransferase-independent histone chaperone function of Dot1 (29). Therefore, it is likely that the stimulatory effect of Dot1 on H2Bub1 involves interactions with histones or the nucleosome or acts via histone dynamics during transcription to subsequently affect enzymes that act on H2BK123. Cellular H2Bub1 levels reflect the balance of ubiquitin ligase and deubiquitinase activities. The genetic interactions between Dot1 overexpression and *ubp8Δ* (Figures 1 and 2) suggest that Ubp8 and Dot1 make independent contributions. Ubp10 is a second deubiquitinase that removes H2Bub1, but Dot1 can increase H2Bub1 independent of Ubp8 as well as Ubp10 (Figure 1). Therefore, it is not likely that Dot1 increases H2Bub1 by interfering with the activity of Ubp8 or Ubp10. Our results show that the ubiquitin ligase Bre1 is required for the increase in H2BK123ub1 by Dot1 overexpression, and that Dot1 acts downstream of Paf1 and hence downstream of transcription elongation. Therefore, we favor the hypothesis that Dot1 enhances H2BK123ub1 by promoting the synthesis of H2BK123ub1 by Bre1 (Figure 4E). Recent elegant studies from the Köhler lab showed that Bre1 interacts with the acidic patch on the surface of the nucleosome and has multiple interactions with the ubiquitin-conjugating enzyme Rad6 to position it on the nucleosome in order to align with K123 and promote the transfer of ubiquitin (57,58). Interestingly, SAGA-DUB also binds to the nucleosome acidic patch and competes with Bre1 for binding to the nucleosome (57–59). However, mutations of the acidic patch do not affect Bre1 and SAGA-DUB equally (57), suggesting that the acidic patch provides opportunities for regulating competing activities and shifting the balance between ubiquitin synthesis and removal. It is tempting to speculate that Dot1 might be one such factor that specifically promotes H2BK123ub1 synthesis, but this idea requires further study.

Finally, the histone H3K4 methyltransferase activities of MLL3/MLL4 and Set1a have recently been shown to be dispensable for facilitating enhancer activity and embryonic stem cell (ESC) self-renewal, respectively (60,61). In addition, methyltransferase-independent functions have recently been uncovered for the histone H3K9 methyltransferase G9a, the H4K20 methyltransferase PR-Set7 and the H3K4 methyltransferase SETD1A (62–64). Our finding that Dot1 can promote H2B ubiquitination independent of H3K79 methylation adds to the growing body of evidence that histone methyltransferases can harbor important catalytic activity-independent regulatory functions in chromatin.

## DATA AVAILABILITY

The deep sequencing data have been deposited in GEO with accession number GSE109144.

## Supplementary Material

Supplementary Data
