# Supplementary material for: Dot1 promotes H2B ubiquitination by a methyltransferase-independent mechanism
Source: Nucleic Acids Res. 2018 Sep 8;46(21):11251–61. doi: 10.1093/nar/gky801 (PMC6265471; doi:10.1093/nar/gky801)
Supplement: Supplementary Data [file gky801_supplemental_files.zip › VanWelsem_Supp_gky801_180906.pdf]

Supplemental Figures

Van Welsem et al, Dot1 promotes H2B ubiquitination by a methyltransferase-independent mechanism

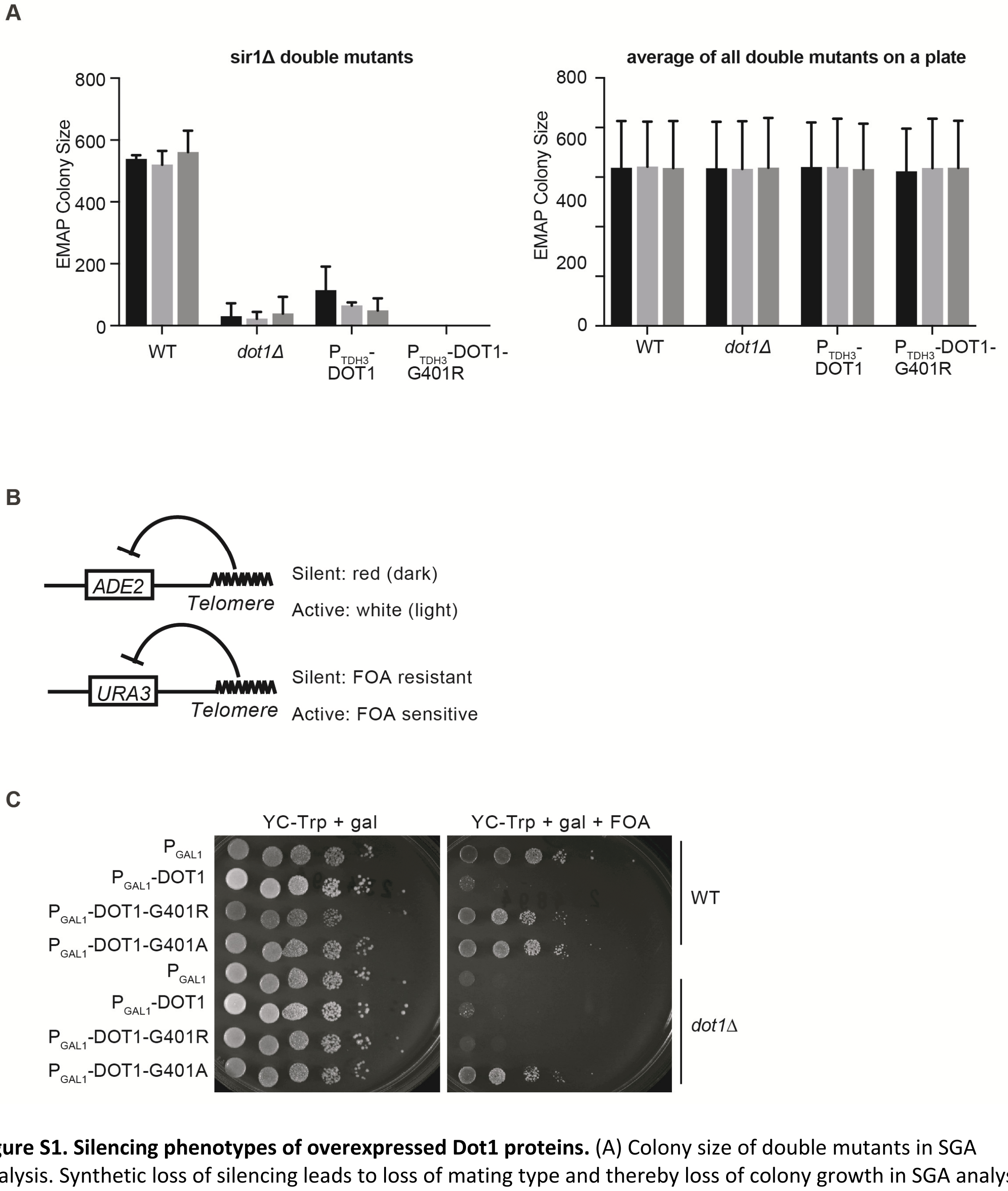

Figure S1. Silencing phenotypes of overexpressed Dot1 proteins. (A) Colony size of double mutants in SGA analysis. Synthetic loss of silencing leads to loss of mating type and thereby loss of colony growth in SGA analyses. WT refers to a control strain harboring a NatMX selectable marker at the intergenic region downstream of the telomeric *ADH4* gene. Left panel shows average colony sizes of indicated alleles combined with *sir1Δ;* right panel shows average colony sizes of all the colonies on a plate as a reference and confirming that the single query alleles by themselves showed no general silencing defects (n=2 ± SD). Black is untreated, light grey 20 J/m^2, dark grey 80 J/m^2 UV irradiation. (B) Outline of the silencing assays using reporter genes. (C) Overexpression of Dot1 disrupted telomeric silencing, whereas Dot1-G401R had no effect. Overexpression of a hypomorphic Dot1-G401A mutant complemented a *dot1Δ.*

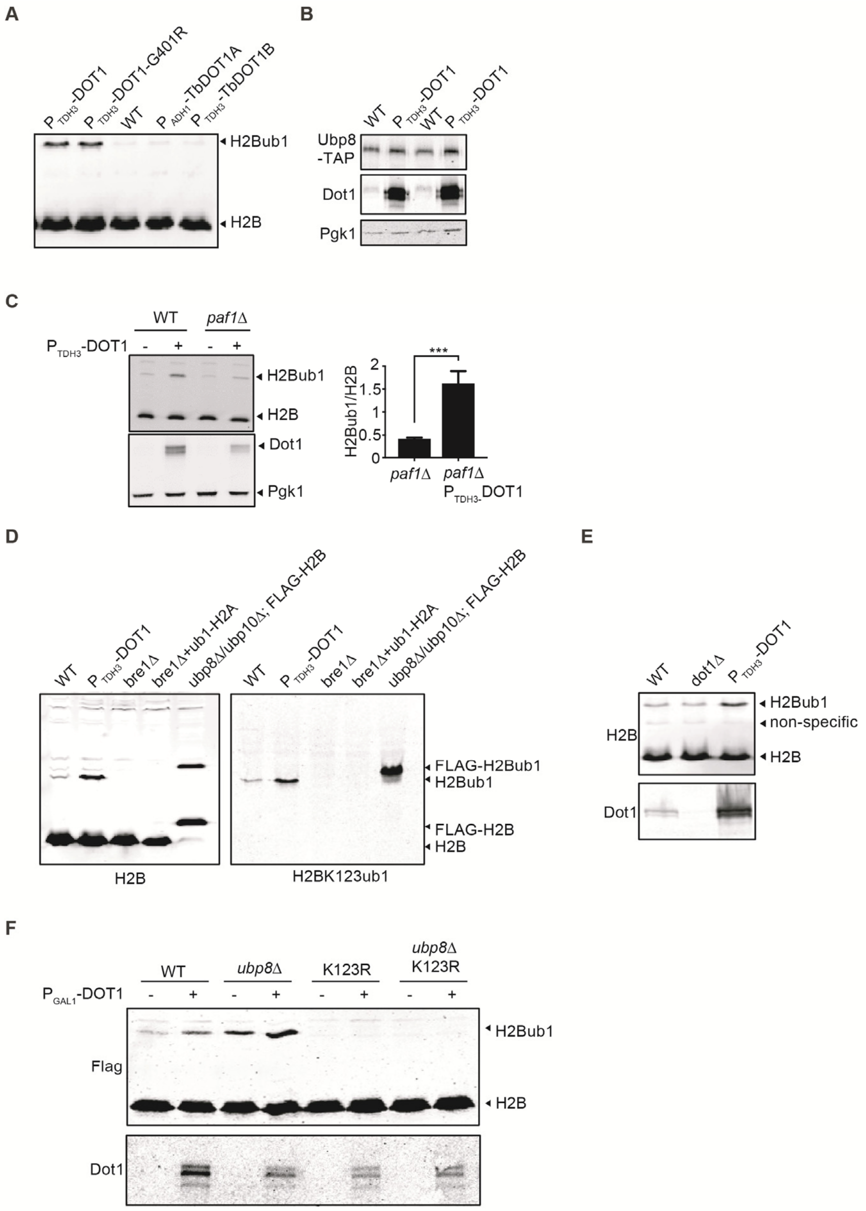

Figure S2. H2BK123ub1 monoclonal antibody. (A) Overexpression of Dot1 proteins from T. brucei led to nearly full H3K79 methylation (1) but has no effect on H2Bub1. (B) Immunoblot analysis of expression of TAP-tagged Ubp8 in the absence of presence of constitutive overexpression of Dot1. (C) Immunoblot analysis showing H2Bub1 levels in a WT and *paf1Δ* strain with or without constitutive overexpression of Dot1. Barplot shows the perecent H2Bub1/H2B (average ± SD of three biological replicates). Statistical significance as determined by a unpaired t-test is indicated by the asterisks (**** $p<0.001$). (D) Immunoblot of control samples to validate the H2BK123ub1 antibody using strains harboring wild-type levels (WT), increased levels (P_{TDH3}*-DOT1*), or no H2BK123ub1 (*bre1Δ*), or ubiquitin fused to H2A (ub1-H2A), or increased levels of H2BK123ub1 on a FLAG-tagged version of H2B (*ubp8Δ;ubp10Δ; FLAG-H2B*). The blot on the left was probed with an H2B antibody, the blot on the right was probed with the monoclonal antibody against H2BK123ub described in this manuscript. (E) Immunoblot analysis of H2Bub1 levels in WT, *dot1Δ,* and Dot1-OE strains. (F) Immunoblot analysis Dot1-OE in *ubp8Δ* an H2BK123R mutant strains shown in Figure 2H. Dot1 was overexpressed in the strains indicated from a galactose-inducible *GAL1* promoter on a multi-copy (2μ) plasmid (+) and an empty vector was used as a negative control (-).

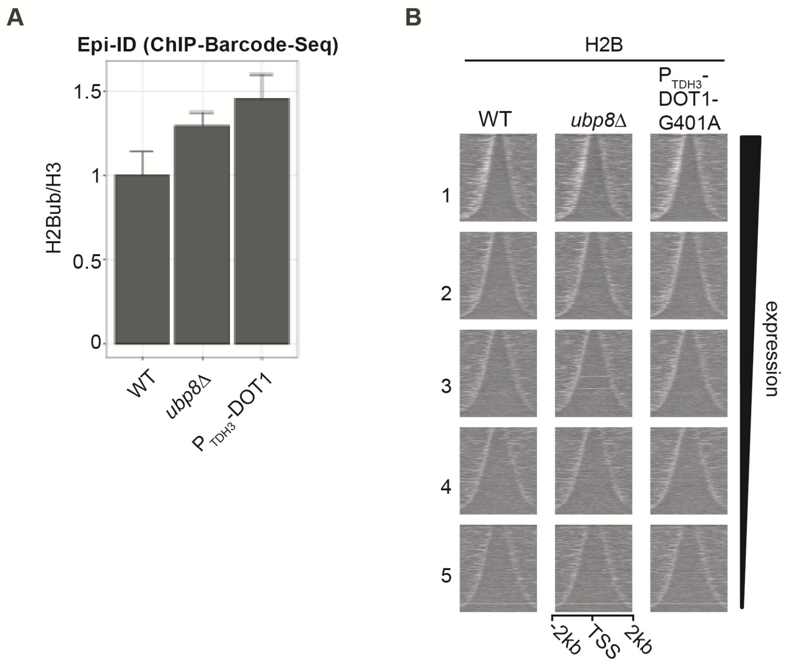

Figure S3. (A) Small-scale H2BK123ub1 Epi-ID experiment. ChIP-barcode-seq on a previously described set of pooled mutants (Epi-ID; see (2)) confirmed that overexpression of Dot1 and deletion of Ubp8 led to more H2BK123ub1 in chromatin at a barcoded *HO* locus. (B) Heatmaps of read-depth normalized H2B ChIP-seq counts showing the H2B signal in the five different gene-expression groups indicated in panel B of Figure 3. Genes within each subgroup were ranked on gene length and centered on the gene midpoint.

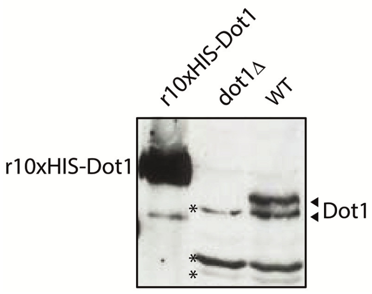

Figure S4. Characterization of the polyclonal pan-Dot1 antiserum. The antiserum detects purified recombinant Dot1 (10xHIS tag) and endogenously expressed Dot1, which is expressed a long and a short isoform. One of the non-specific bands (asterisks) co-migrates with the shorter isoform of Dot1 in whole-cell yeast extracts.

Table S1. Yeast strains used in this study

Strain	Genotype	Figure	ref
Y7092	MATα can1Δ::STE2pr-Sp_his5 lyp1Δ his3Δ1 leu2Δ0 ura3Δ0 met15Δ0	1A-C, S2C+E, Table S5	(3)
NKI2052	MATα can1Δ::STE2pr-Sp_his5 lyp1Δ his3Δ1 leu2Δ0 ura3Δ0 met15Δ0 dot1::NatMX	1A, S2E, Table S5	(4)
NKI4553	MATα can1Δ::STE2pr-Sp_his5 lyp1Δ his3Δ1 leu2Δ0 ura3Δ0 met15Δ0 dot1::NatNT2-TDH3pr-DOT1	1A-C, S2A+C-E, Table S5	(2)
NKI2378	MATα can1Δ::STE2pr-Sp_his5 lyp1Δ his3Δ1 leu2Δ0 ura3Δ0 met15Δ0 dot1::NatNT2-TDH3pr-DOT1G401R	1A-C, S2A, Table S5	This study
NKI2527	MATα can1Δ::STE2pr-Sp_his5 lyp1Δ his3Δ1 leu2Δ0 ura3Δ0 met15Δ0 paf1::KanMX	S2C	This study
NKI2528	MATα can1Δ::STE2pr-Sp_his5 lyp1Δ his3Δ1 leu2Δ0 ura3Δ0 met15Δ0 dot1::NatNT2-TDH3pr-DOT1 paf1::KanMX	S2C	This study
UCC7315	MATa lys2Δ0 trp1Δ63 his3Δ200 ade2::hisG ura3Δ0 leu2Δ0 met15Δ0 hta1-htb1::MET15 hta2-htb2::LEU2 ADE2-TEL-VR URA3-TEL-VIIL pCEN-LYS2-HTA1-HTB1 pCS1 (pCEN-LYS2-HTA1-HTB1)	1D, 2A-B	(5)
UCC6288	MATa lys2Δ0 trp1Δ63 his3Δ200 ade2::hisG ura3Δ0 leu2Δ0 met15Δ0 hta1-htb1::MET15 hta2-htb2::LEU2 ADE2-TEL-VR URA3-TEL-VIIL ubp8::KanMX pCS1 (pCEN-LYS2-HTA1-HTB1)	1D, 2A-B	(5)
NKI4625	MATa lys2Δ0 trp1Δ63 his3Δ200 ade2::hisG ura3Δ0 leu2Δ0 met15Δ0 hta1-htb1::MET15 hta2-htb2::LEU2 bre1::KanMX pJH23 (pCEN-HIS3-HTA1-HTB1)	2A-B	(6)
UCC6361	MATa lys2Δ0 trp1Δ63 his3Δ200 ade2::hisG ura3Δ0 leu2Δ0 met15Δ0 hta1-htb1::MET15 hta2-htb2::LEU2 ADE2-TEL-VR URA3-TEL-VIIL pCS1 ubp10::NatMX pCS1 (pCEN-LYS2-HTA1-HTB1)	2A-B	(7)
NKI4609	MATa lys2Δ0 trp1Δ63 his3Δ200 ura3Δ0 leu2Δ0 met15Δ0 hta1-htb1::MET15 hta2-htb2::LEU2 pRG422 (pCEN-HIS3-HTA1-FLAG-HTB1)	2C	(2)
NKI4610	MATa lys2Δ0 trp1Δ63 his3Δ200 ura3Δ0 leu2Δ0 met15Δ0 hta1-htb1::MET15 hta2-htb2::LEU2 pRG423 (pCEN-HIS3-HTA1-FLAG-HTB1-K123R)	2C	(2)
NKI4560	MATa can1Δ::STE2pr-Sp_his5 lyp1Δ his3Δ1 leu2Δ0 ura3Δ0 met15Δ0 ho::BC-KanMX	2D	(2)
NKI4558	MATa can1Δ::STE2pr-Sp_his5 lyp1Δ his3Δ1 leu2Δ0 ura3Δ0 met15Δ0 ho::BC-KanMX bre1::NatMX	2D, S2D	(2)
NKI3031	MATa leu2Δ0 lys2Δ0 ura3Δ0	2F-G	(1)
NKI8040	MATa leu2Δ0 lys2Δ0 ura3Δ0 KanMX-TDH3pr-5'UTR-DOT1	2F-G	(1)
NKI6109	MATa leu2Δ0 lys2Δ0 ura3Δ0 NatNT2-ADH1pr-DOT1	2F-G	(1)
NKI8038	MATa leu2Δ0 lys2Δ0 ura3Δ0 KanMX-TDH3pr-DOT1	2F-G	(1)
NKI8039	MATa leu2Δ0 lys2Δ0 ura3Δ0 KanMX-TEF1pr-DOT1	2F-G	(1)
KY2513	MATα his3Δ200 lysΔ2-128 Leu2Δ1 uraΔ3-52 FLAG-BRE1	2E	(2)
NKI2544	MATα his3Δ200 lysΔ2-128 Leu2Δ1 uraΔ3-52 FLAG-BRE1 dot1::NatMX	2E	This study
NKI2545	MATα his3Δ200 lysΔ2-128 Leu2Δ1 uraΔ3-52 FLAG-BRE1 NatNT2-TDH3prom-DOT1	2E	This study
NKI6061	MATa his3Δ1 leu2Δ0 lys2Δ0 met15Δ0 trp1Δ63 ura3Δ0 arg4::KanMX	3A-C, 4B, S2A, S3B	(1)
NKI6142	MATa his3Δ1 leu2Δ0 lys2Δ0 met15Δ0 trp1Δ63 ura3Δ0 arg4::KanMX bre1::HphMX	3A	This study
NKI4656	MATa can1d::STE2pr-Sp_his5 lyp1d his3d1 leu2d0 ura3d0 met15d0 ho::BC-KanMX-BC ubp8::NatMX	3A-C, S3B	(2)

NKI6152	MATa his3Δ1 leu2Δ0 lys2Δ0 met15Δ0 trp1Δ63 ura3Δ0 arg4::KanMX dot1::NatNT2-TDH3pr-DOT1G-401A	3A-C, 4B, S3B	This study
NKI6151	MATa his3Δ1 leu2Δ0 lys2Δ0 met15Δ0 trp1Δ63 ura3Δ0 arg4::KanMX URA3-TDH3pr-Dot1	4B	(1)
NKI6153	MATa his3Δ1 leu2Δ0 lys2Δ0 met15Δ0 trp1Δ63 ura3Δ0 arg4::KanMX dot1::NatMX-TDH3pr-Dot1-Δ2-172	4B	This study
BY4733	MATa his3Δ200 leu2Δ0 met15Δ0 trp1Δ63 ura3Δ0	4C-D	(8)
UCC7366	MATa lys2Δ0 trp1Δ63 his3Δ200 ade2::hisG ura3Δ0 leu2Δ0 met15Δ0 ADE2-TEL-VR URA3-TEL-VIIL	S1C	(4)
UCC7356	MATa lys2Δ0 trp1Δ63 his3Δ200 ade2::hisG ura3Δ0 leu2Δ0 met15Δ0 ADE2-TEL-VR URA3-TEL-VIIL dot1::NatMX	S1C	(9)
NKI6114	MATa his3Δ1 leu2Δ0 lys2Δ0 met15Δ0 trp1Δ63 ura3Δ0 arg4::KanMX dot1::NatMX-ADH1pr-TbDOT1A HMLα1/2Δ::HphMX	S2A	(1)
NKI6128	MATa his3Δ1 leu2Δ0 lys2Δ0 met15Δ0 trp1Δ63 ura3Δ0 arg4::KanMX dot1:: NatNT2-TDH3pr-TbDOT1B HMLα1/2Δ::HphMX	S2A	(1)
BY4741	MATa his3Δ1 leu2Δ0 met15Δ0 ura3Δ0	S2D	(8)
BY4742	MATα his3Δ1 leu2Δ0 lys2Δ0 ura3Δ0	Table S4	(8)
NKI3002	MATα his3Δ1 leu2Δ0 lys2Δ0 ura3Δ0 dot1Δ::KanMX	Table S4	This study
NKI8046	MATα his3Δ1 leu2Δ0 lys2Δ0 ura3Δ0 KanMX-TDH3pr-DOT1	Table S4	This study
NKI8047	MATα his3Δ1 leu2Δ0 lys2Δ0 ura3Δ0 KanMX-TEF1pr-DOT1	Table S4	This study
NKI8048	MATα his3Δ1 leu2Δ0 lys2Δ0 ura3Δ0 KanMX-TDH3pr-DOT1-G401V	Table S4	This study
NKI8049	MATα his3Δ1 leu2Δ0 lys2Δ0 ura3Δ0 KanMX-TEF1pr-DOT1-G401V	Table S4	This study
NKI4628	MATa lys2Δ0 trp1Δ63 his3Δ200 ura3Δ0 leu2Δ0 met15Δ0 hta1-htb1Δ::MET15 hta2-htb2Δ::LEU2 bre1Δ::KanMX pHV021 (pCEN-HIS3-ub-HA-S1-HTA1-HTB1)	S2D	(6)
UCC6393	MATa lys2Δ0 trp1Δ63 his3Δ200 ade2::hisG ura3Δ0 leu2Δ0 met15Δ0 hta1-htb1::MET15 hta2-htb2::LEU2 ADE2-TEL-VR URA3-TEL-VIIL ubp8::KanMX ubp10::NatMX pRG422 (pCEN-HIS3-HTA1-FLAG-HTB1)	S2D	Gift from Richard Gardner
NKI3027	MATa lys2Δ0 trp1Δ63 his3Δ200 ura3Δ0 leu2Δ0 met15Δ0 ade2::hisG ADE2-TEL-VR URA3-TEL-VIIL hta1-htb1::MET15 hta2-htb2::LEU2 pRG422 (pCEN-HIS3-HTA1-FLAG-HTB1)	2H, S2F	This study
NKI3028	MATa lys2Δ0 trp1Δ63 his3Δ200 ura3Δ0 leu2Δ0 met15Δ0 ade2::hisG ADE2-TEL-VR URA3-TEL-VIIL hta1-htb1::MET15 hta2-htb2::LEU2 pRG423 (pCEN-HIS3-HTA1-FLAG-HTB1-K123R)	2H, S2F	This study
NKI2563	MATa lys2Δ0 trp1Δ63 his3Δ200 ura3Δ0 leu2Δ0 met15Δ0 ade2::hisG ADE2-TEL-VR URA3-TEL-VIIL hta1-htb1::MET15 hta2-htb2::LEU2 ubp8::KanMX pRG422 (pCEN-HIS3-HTA1-FLAG-HTB1)	2H, S2F	This study
NKI2564	MATa lys2Δ0 trp1Δ63 his3Δ200 ura3Δ0 leu2Δ0 met15Δ0 ade2::hisG ADE2-TEL-VR URA3-TEL-VIIL hta1-htb1::MET15 hta2-htb2::LEU2 ubp8::KanMX pRG423 (pCEN-HIS3-HTA1-FLAG-HTB1-K123R)	2H, S2F	This study
NKI4748	MATα can1Δ::HphMX lyp1Δ::STE3pr-LEU2 his3Δ1 leu2Δ0 ura3Δ0 met15Δ0 ho::BC-KanMX-BC UBP8-TAP-His3MX6	S2B	This study
NKI2566	MATα can1Δ::HphMX lyp1Δ::STE3pr-LEU2 his3Δ1 leu2Δ0 ura3Δ0 met15Δ0 ho::BC-KanMX-BC UBP8-TAP-His3MX6 NatNT2-TDH3pr-DOT1	S2B	This study

Table S2. Plasmids used in this study

Plasmid	Figure	Description	Reference
pTCG	1D, 2A-D, 2H, 4C-D, S1C, S2A, S2F	pGAL1 (2µ-TRP1)	(10)
pFvL018	1D, 2A-D, 2H, 4C-D, S1C, S2A, S2F	pGAL1-Dot1	(10,11)
pFvL043	2C, S1C, S2A	pGAL1-Dot1-G401R	(1,10)
pFvL044	S1C	pGAL1-Dot1-G401A	(10)
pFvL019	4C	pGAL1-Dot1-1-172	This study
pRG422pRG423	see strain table	pCEN-HIS3-HTA1-FLAG-HTB1-K123R	(10)

Table S3. Oligos used for qPCR

Name	Sequence	Amplicon (bp)	Chromosome - position
SPA2_MidLow_Qfor	GAGAAGGGCGCATCTGTAGAAT	64	chrXII:103527-103590
SPA2_Midlow_Qrev	CGACATTACCAAGTGGTTCTTGAA		
FMP27_PRO_Qfor1	AGGGAGACATGAAAAAGGGTCTT	107	chrXII:1043789-1043895
FMP27_PRO_Qrev1	TCTCTGAGATGTCTAGGCCCTTTTA		
PMA1_PRO_Qfor1	TGGTGGGTACCGCTTATGCT	97	chrVII:482946-483042
PMA1_PRO_Qrev1	TGTTAGACGATAATGATAGGACATTTGA		
NoORF_Qfor	GGCTGTCAGAATATGGGGCCGTAGTA	148	chrV:9716-9863
NoORF_Qrev	CACCCCGAAGCTGCTTTCACAATAC		

Supplemental References

1. Stulemeijer, I.J.E., De Vos, D., van Harten, K., Joshi, O.K., Blomberg, O., van Welsem, T., Terweij, M., Vlaming, H., de Graaf, E.L., Altelaar, A.F.M. *et al.* (2015) Dot1 histone methyltransferases share a distributive mechanism but have highly diverged catalytic properties. *Sci. Rep.*, **5**, 9824.
2. Vlaming, H., Molenaar, T.M., van Welsem, T., Poramba-Liyanage, D.W., Smith, D.E., Velds, A., Hoekman, L., Korthout, T., Hendriks, S., Altelaar, A.M. *et al.* (2016) Direct screening for chromatin status on DNA barcodes in yeast delineates the regulome of H3K79 methylation by Dot1. *eLife*, **5**, 10.7554/eLife.18919.
3. Tong, A.H. and Boone, C. (2006) Synthetic genetic array analysis in Saccharomyces cerevisiae. *Methods Mol. Biol.*, **313**, 171-192.
4. van Welsem, T., Frederiks, F., Verzijlbergen, K.F., Faber, A.W., Nelson, Z.W., Egan, D.A., Gottschling, D.E. and van Leeuwen, F. (2008) Synthetic lethal screens identify gene silencing processes in yeast and implicate the acetylated amino terminus of Sir3 in recognition of the nucleosome core. *Mol. Cell. Biol.*, **28**, 3861-3872.
5. Gardner, R.G., Nelson, Z.W. and Gottschling, D.E. (2005) Ubp10/Dot4p regulates the persistence of ubiquitinated histone H2B: distinct roles in telomeric silencing and general chromatin. *Mol. Cell. Biol.*, **25**, 6123-6139.
6. Vlaming, H., van Welsem, T., de Graaf, E.L., Ontoso, D., Altelaar, A.M., San-Segundo, P.A., Heck, A.J. and van Leeuwen, F. (2014) Flexibility in crosstalk between H2B ubiquitination and H3 methylation in vivo. *EMBO Rep.*, **15**, 1077-1084.
7. Gardner, R.G., Nelson, Z.W. and Gottschling, D.E. (2005) Degradation-mediated protein quality control in the nucleus. *Cell*, **120**, 803-815.
8. Brachmann, C.B., Davies, A., Cost, G.J., Caputo, E., Li, J., Hieter, P. and Boeke, J.D. (1998) Designer deletion strains derived from Saccharomyces cerevisiae S288C: a useful set of strains and plasmids for PCR-mediated gene disruption and other applications. *Yeast*, **14**, 115-132.
9. Verzijlbergen, K.F., Menendez-Benito, V., van Welsem, T., van Deventer, S.J., Lindstrom, D.L., Ovaa, H., Neefjes, J., Gottschling, D.E. and van Leeuwen, F. (2010) Recombination-induced tag exchange to track old and new proteins. *Proc. Natl. Acad. Sci. U.S.A.*, **107**, 64-68.
10. Frederiks, F., Tzouros, M., Oudgenoeg, G., van Welsem, T., Fornerod, M., Krijgsveld, J. and van Leeuwen, F. (2008) Nonprocessive methylation by Dot1 leads to functional redundancy of histone H3K79 methylation states. *Nat. Struct. Mol. Biol.*, **15**, 550-557.
11. van Leeuwen, F., Gafken, P.R. and Gottschling, D.E. (2002) Dot1p modulates silencing in yeast by methylation of the nucleosome core. *Cell*, **109**, 745-756.
